# *cis*-Bis[2-(1,3-benzothia­zol-2-yl)-1-(4-fluoro­phen­yl)ethen­yl](pentane-2,4-dionato-κ^2^ *O*,*O*′)iridium(III)

**DOI:** 10.1107/S1600536809010204

**Published:** 2009-03-25

**Authors:** Guo-Yong Xiao, Peng Lei, Hai-Jun Chi, Zhi-Zhi Hu, Xiao Li

**Affiliations:** a School of Chemical Engineering, University of Science and Technology Liaoning, Anshan 114051, People’s Republic of China; b Key Laboratory of Excited State Processes, Changchun Institute of Optics, Fine Mechanics and Physics, Chinese Academy of Sciences, Changchun 130033, People’s Republic of China

## Abstract

In the title compound, [Ir(C_15_H_9_FNS)_2_(C_5_H_7_O_2_)], the Ir atom is hexa­coordinated by three chelating ligands, with two cyclo­metalated 2-(1,3-benzothia­zol-2-yl)-1-(4-fluoro­phen­yl)ethenyl ligands showing *N*,*C*-bidentate coordination and an *O*,*O*′-bidenate pentane-2,4-dionate anion, thereby forming a distorted octa­hedral enviroment.

## Related literature

For a related structure, see: Li *et al.* (2008 ▶). For background to possible applications of this class of compound, see: Baldo *et al.* (1998 ▶); Forrest (2003 ▶).
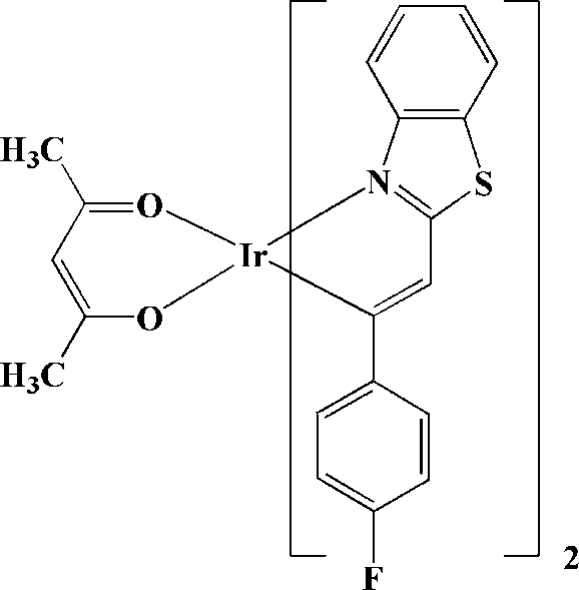


## Experimental

### 

#### Crystal data

[Ir(C_15_H_9_FNS)_2_(C_5_H_7_O_2_)]
*M* *_r_* = 799.89
Monoclinic, 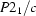

*a* = 9.1632 (18) Å
*b* = 17.736 (4) Å
*c* = 18.823 (4) Å
β = 93.06 (3)°
*V* = 3054.7 (11) Å^3^
*Z* = 4
Mo *K*α radiation
μ = 4.56 mm^−1^
*T* = 113 K
0.16 × 0.14 × 0.10 mm

#### Data collection

Rigaku Saturn diffractometer
Absorption correction: multi-scan (*SADABS*; Sheldrick, 1996 ▶) *T*_min_ = 0.529, *T*_max_ = 0.659
20289 measured reflections
5373 independent reflections
4792 reflections with *I* > 2σ(*I*)
*R*_int_ = 0.055

#### Refinement

*R*[*F* ^2^ > 2σ(*F* ^2^)] = 0.029
*wR*(*F* ^2^) = 0.069
*S* = 1.05
5373 reflections
399 parameters
H-atom parameters constrained
Δρ_max_ = 1.37 e Å^−3^
Δρ_min_ = −2.58 e Å^−3^

### 

Data collection: *CrystalClear* (Rigaku, 1999 ▶); cell refinement: *CrystalClear*; data reduction: *CrystalClear*; program(s) used to solve structure: *SHELXS97* (Sheldrick, 2008 ▶); program(s) used to refine structure: *SHELXL97* (Sheldrick, 2008 ▶); molecular graphics: *SHELXTL* (Sheldrick, 2008 ▶); software used to prepare material for publication: *SHELXTL*.

## Supplementary Material

Crystal structure: contains datablocks global, I. DOI: 10.1107/S1600536809010204/hb2924sup1.cif

Structure factors: contains datablocks I. DOI: 10.1107/S1600536809010204/hb2924Isup2.hkl

Additional supplementary materials:  crystallographic information; 3D view; checkCIF report

## Figures and Tables

**Table 1:** Selected bond lengths (Å)

Ir1—C9	2.000 (4)
Ir1—C24	1.988 (4)
Ir1—N1	2.045 (3)
Ir1—N2	2.049 (3)
Ir1—O1	2.137 (2)
Ir1—O2	2.137 (3)

**Fig. 1.**
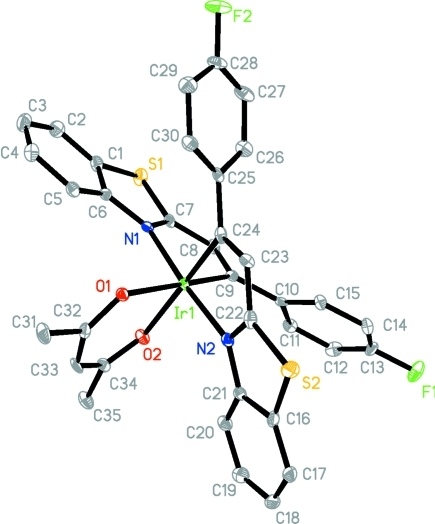
View of the molecule of (I) with displacement ellipsoids drawn at the 35% probability level. The H atoms are omitted for clarity.

## supplementary crystallographic information

### Comment

Organic triplet-state light-emitting materials (organic phosphorophores) have been one of the most important recent develophments in the field of organic light-emitting diodes (OLEDs) (Baldo *et al.*, 1998; Forrest, 2003). we now report the crystal structure of the title compound, (I), a new iridium(III) complex with benzothiazole and acetylacetonate ligands. The atomic connectivity of (I) was elucidated by extensive spectroscopic analysis, including two-dimensional NMR spectroscopy, and confirmed by single-crystal X-ray diffraction analysis (Fig. 1)

The title compound is a netural mononuclear iridium(III) complex. All the bond lengths and angles fall within their normal ranges. The iridium centre is coordinated by two N atoms and two C atoms from the two 2-(4-fluorostyryl)benzo[*d*]thiazole anions and two O atoms for the β-diketonate (Table 1). The Ir—C bond lengths [1.988 (4) and 2.000 (4) Å] are found to be shorter than the Ir—N bonds [2.045 (3) and 2.049 Å], as seen in related compounds (Li *et al.*,2008). The two five-numbered chelate rings are nearly coplanar with the r.m.s. deviations of 0.0549 (3) for C7—C8—C9—N1—Ir1 and 0.0705 (3)Å for C22—C23—C24—N2—Ir1. The dihedral angles between the two benzo[*d*]thiazoles and two fluorobenzene rings are 59.2 (2) and 84.9 (2)°, respectively, which indicates that two fluorobenzene units are almost perpendicular.

### Experimental

The title compound was prepared by the reaction of (*E*)-2-(4-fluorostyryl)benzothiazole (2.2 mmol) in 2-ethoxyethaol (10 mL) with iridium trichloride hydrate (1.0 mmol) in 3.0 ml of water for 12 h at 353 K. The crude product was purified on a silica gel column using acetic ether and n-hexane as eluent to give the desired red powder of the target compound in 42% yield. Red prisms of (I) were grown by slow evaporation of a solution in methylene chloride/methanol(1:3). Spectroscopic analysis: ^1^H NMR (500 MHz, CDCl_3_, p.p.m.): 1.71 (s, 6H), 6.41 (t, 4H), 6.78 (t, 4H), 7.00–7.08 (m, 6H), 7.31 (d, 2H), 7.53 (d, 2H). MS APCI (m/*z*): 800.9 [*M*+1]^+^.

### Refinement

All H atoms were positioned geometrically and refined as riding (C—H = 0.93–0.96 Å) and allowed to ride on their parent atoms, with *U*_iso_(H) = 1.2*U*_eq_(parent) or 1.5*U*_eq_(parent).

### Crystal data

**Table:**

[Ir(C_15_H_9_FNS)_2_(C_5_H_7_O_2_)]	*F*(000) = 1568
*M**_r_* = 799.89	*D*_x_ = 1.739 Mg m^−^^3^
Monoclinic, *P*2_1_/*c*	Mo *K*α radiation, λ = 0.71073 Å
Hall symbol: -P 2ybc	Cell parameters from 10390 reflections
*a* = 9.1632 (18) Å	θ = 1.6–27.9°
*b* = 17.736 (4) Å	µ = 4.56 mm^−^^1^
*c* = 18.823 (4) Å	*T* = 113 K
β = 93.06 (3)°	Prism, red
*V* = 3054.7 (11) Å^3^	0.16 × 0.14 × 0.10 mm
*Z* = 4	

### Data collection

**Table:**

Rigaku Saturn diffractometer	5373 independent reflections
Radiation source: rotating anode	4792 reflections with *I* > 2σ(*I*)
confocal	*R*_int_ = 0.055
ω scans	θ_max_ = 25.0°, θ_min_ = 1.6°
Absorption correction: multi-scan (*SADABS*; Sheldrick, 1996)	*h* = −10→9
*T*_min_ = 0.529, *T*_max_ = 0.659	*k* = −21→20
20289 measured reflections	*l* = −22→21

### Refinement

**Table:**

Refinement on *F*^2^	Primary atom site location: structure-invariant direct methods
Least-squares matrix: full	Secondary atom site location: difference Fourier map
*R*[*F*^2^ > 2σ(*F*^2^)] = 0.029	Hydrogen site location: inferred from neighbouring sites
*wR*(*F*^2^) = 0.069	H-atom parameters constrained
*S* = 1.05	*w* = 1/[σ^2^(*F*_o_^2^) + (0.0328*P*)^2^] where *P* = (*F*_o_^2^ + 2*F*_c_^2^)/3
5373 reflections	(Δ/σ)_max_ = 0.005
399 parameters	Δρ_max_ = 1.37 e Å^−^^3^
0 restraints	Δρ_min_ = −2.58 e Å^−^^3^

### Special details

**Table:**

Geometry. All e.s.d.'s (except the e.s.d. in the dihedral angle between two l.s. planes) are estimated using the full covariance matrix. The cell e.s.d.'s are taken into account individually in the estimation of e.s.d.'s in distances, angles and torsion angles; correlations between e.s.d.'s in cell parameters are only used when they are defined by crystal symmetry. An approximate (isotropic) treatment of cell e.s.d.'s is used for estimating e.s.d.'s involving l.s. planes.
Refinement. Refinement of *F*^2^ against ALL reflections. The weighted *R*-factor *wR* and goodness of fit *S* are based on *F*^2^, conventional *R*-factors *R* are based on *F*, with *F* set to zero for negative *F*^2^. The threshold expression of *F*^2^ > σ(*F*^2^) is used only for calculating *R*-factors(gt) *etc*. and is not relevant to the choice of reflections for refinement. *R*-factors based on *F*^2^ are statistically about twice as large as those based on *F*, and *R*- factors based on ALL data will be even larger.

### Fractional atomic coordinates and isotropic or equivalent isotropic displacement parameters (Å^2^)

**Table:**

	*x*	*y*	*z*	*U*_iso_*/*U*_eq_	
Ir1	0.914147 (14)	0.885461 (7)	0.233249 (7)	0.01190 (7)	
S1	0.66028 (11)	1.04464 (5)	0.36383 (6)	0.0239 (2)	
S2	1.28983 (11)	0.75043 (6)	0.16085 (6)	0.0256 (3)	
F1	1.5245 (3)	1.09581 (13)	0.14514 (15)	0.0368 (7)	
F2	0.9677 (3)	0.84075 (14)	0.60215 (13)	0.0403 (7)	
O1	0.8070 (3)	0.77954 (13)	0.21589 (14)	0.0151 (6)	
O2	0.7795 (3)	0.92998 (13)	0.14703 (14)	0.0182 (6)	
N1	0.7667 (3)	0.93021 (16)	0.29937 (16)	0.0133 (7)	
N2	1.0741 (3)	0.84154 (16)	0.17349 (17)	0.0158 (7)	
C1	0.5810 (4)	0.9564 (2)	0.3751 (2)	0.0206 (9)	
C2	0.4627 (4)	0.9380 (2)	0.4159 (2)	0.0265 (10)	
H2	0.4141	0.9749	0.4405	0.032*	
C3	0.4204 (4)	0.8629 (2)	0.4183 (2)	0.0290 (11)	
H3	0.3431	0.8489	0.4454	0.035*	
C4	0.4931 (4)	0.8086 (2)	0.3802 (2)	0.0259 (10)	
H4	0.4628	0.7586	0.3826	0.031*	
C5	0.6090 (4)	0.8264 (2)	0.3390 (2)	0.0193 (9)	
H5	0.6559	0.7894	0.3137	0.023*	
C6	0.6529 (4)	0.9014 (2)	0.3367 (2)	0.0159 (9)	
C7	0.7865 (4)	1.0049 (2)	0.3091 (2)	0.0175 (9)	
C8	0.9091 (4)	1.0382 (2)	0.2803 (2)	0.0169 (9)	
H8	0.9285	1.0896	0.2836	0.020*	
C9	0.9980 (4)	0.9889 (2)	0.2469 (2)	0.0149 (8)	
C10	1.1375 (4)	1.01596 (19)	0.21908 (19)	0.0140 (8)	
C11	1.1421 (4)	1.0838 (2)	0.1814 (2)	0.0186 (9)	
H11	1.0570	1.1119	0.1737	0.022*	
C12	1.2707 (5)	1.10982 (19)	0.1553 (2)	0.0233 (10)	
H12	1.2725	1.1540	0.1288	0.028*	
C13	1.3955 (4)	1.0689 (2)	0.1695 (2)	0.0221 (9)	
C14	1.3982 (4)	1.0015 (2)	0.2060 (2)	0.0217 (9)	
H14	1.4845	0.9744	0.2137	0.026*	
C15	1.2674 (4)	0.9756 (2)	0.2307 (2)	0.0174 (9)	
H15	1.2661	0.9303	0.2555	0.021*	
C16	1.2342 (4)	0.8054 (2)	0.0874 (2)	0.0212 (9)	
C17	1.2906 (4)	0.8077 (2)	0.0208 (2)	0.0250 (10)	
H17	1.3676	0.7764	0.0098	0.030*	
C18	1.2303 (4)	0.8576 (2)	−0.0292 (2)	0.0273 (10)	
H18	1.2666	0.8597	−0.0743	0.033*	
C19	1.1142 (5)	0.9049 (2)	−0.0119 (2)	0.0252 (10)	
H19	1.0752	0.9386	−0.0457	0.030*	
C20	1.0576 (4)	0.9023 (2)	0.0542 (2)	0.0209 (9)	
H20	0.9808	0.9339	0.0650	0.025*	
C21	1.1164 (4)	0.8520 (2)	0.1048 (2)	0.0167 (9)	
C22	1.1555 (4)	0.7908 (2)	0.2101 (2)	0.0192 (9)	
C23	1.1358 (4)	0.7834 (2)	0.2839 (2)	0.0176 (9)	
H23	1.1867	0.7486	0.3127	0.021*	
C24	1.0345 (4)	0.83243 (19)	0.3087 (2)	0.0175 (9)	
C25	1.0106 (4)	0.83624 (19)	0.3856 (2)	0.0150 (8)	
C26	1.0432 (5)	0.9016 (2)	0.4245 (2)	0.0238 (10)	
H26	1.0747	0.9445	0.4013	0.029*	
C27	1.0292 (5)	0.9029 (2)	0.4973 (2)	0.0272 (10)	
H27	1.0534	0.9460	0.5236	0.033*	
C28	0.9786 (4)	0.8394 (2)	0.5300 (2)	0.0255 (10)	
C29	0.9402 (4)	0.7751 (2)	0.4934 (2)	0.0252 (10)	
H29	0.9022	0.7337	0.5165	0.030*	
C30	0.9599 (4)	0.7738 (2)	0.4208 (2)	0.0205 (9)	
H30	0.9385	0.7299	0.3953	0.025*	
C31	0.6666 (4)	0.6829 (2)	0.1608 (2)	0.0266 (10)	
H31A	0.7460	0.6495	0.1738	0.040*	
H31B	0.6284	0.6709	0.1137	0.040*	
H31C	0.5910	0.6772	0.1937	0.040*	
C32	0.7211 (4)	0.7637 (2)	0.1626 (2)	0.0178 (9)	
C33	0.6716 (4)	0.8130 (2)	0.1087 (2)	0.0200 (9)	
H33	0.6150	0.7918	0.0714	0.024*	
C34	0.6972 (4)	0.89059 (19)	0.1044 (2)	0.0182 (9)	
C35	0.6229 (4)	0.9345 (2)	0.0443 (2)	0.0258 (10)	
H35A	0.5891	0.9818	0.0620	0.039*	
H35B	0.5414	0.9062	0.0244	0.039*	
H35C	0.6910	0.9436	0.0082	0.039*	

### Atomic displacement parameters (Å^2^)

**Table:**

	*U*^11^	*U*^22^	*U*^33^	*U*^12^	*U*^13^	*U*^23^
Ir1	0.01140 (11)	0.01073 (11)	0.01329 (12)	0.00056 (5)	−0.00189 (7)	−0.00079 (5)
S1	0.0190 (6)	0.0185 (5)	0.0348 (7)	0.0027 (4)	0.0051 (4)	−0.0084 (5)
S2	0.0216 (6)	0.0302 (6)	0.0253 (7)	0.0104 (5)	0.0026 (4)	−0.0051 (5)
F1	0.0272 (15)	0.0348 (14)	0.0501 (19)	−0.0077 (12)	0.0173 (12)	0.0091 (13)
F2	0.0611 (19)	0.0461 (16)	0.0138 (15)	0.0079 (13)	0.0026 (12)	−0.0008 (12)
O1	0.0159 (15)	0.0150 (13)	0.0143 (15)	−0.0023 (11)	−0.0009 (11)	0.0011 (11)
O2	0.0196 (15)	0.0139 (14)	0.0204 (16)	0.0014 (12)	−0.0054 (11)	−0.0001 (12)
N1	0.0143 (17)	0.0149 (17)	0.0107 (17)	0.0001 (13)	−0.0013 (12)	−0.0015 (14)
N2	0.0152 (17)	0.0144 (17)	0.0177 (19)	−0.0022 (14)	−0.0011 (13)	−0.0049 (14)
C1	0.016 (2)	0.019 (2)	0.026 (3)	0.0011 (17)	−0.0021 (17)	−0.0025 (18)
C2	0.020 (2)	0.031 (2)	0.029 (3)	0.0056 (19)	0.0019 (18)	−0.004 (2)
C3	0.018 (2)	0.035 (3)	0.034 (3)	0.000 (2)	0.0039 (19)	0.002 (2)
C4	0.020 (2)	0.025 (2)	0.032 (3)	−0.0017 (19)	0.0001 (18)	0.000 (2)
C5	0.017 (2)	0.019 (2)	0.021 (2)	0.0022 (17)	−0.0030 (16)	−0.0006 (17)
C6	0.012 (2)	0.021 (2)	0.014 (2)	0.0031 (17)	−0.0020 (15)	−0.0008 (17)
C7	0.012 (2)	0.022 (2)	0.019 (2)	0.0040 (17)	−0.0044 (15)	−0.0051 (18)
C8	0.018 (2)	0.0093 (19)	0.023 (2)	−0.0016 (16)	−0.0021 (16)	−0.0011 (17)
C9	0.017 (2)	0.016 (2)	0.012 (2)	0.0013 (17)	−0.0036 (15)	0.0020 (16)
C10	0.019 (2)	0.0140 (19)	0.009 (2)	−0.0043 (16)	−0.0004 (15)	−0.0023 (16)
C11	0.021 (2)	0.014 (2)	0.020 (2)	0.0050 (17)	−0.0021 (16)	0.0002 (17)
C12	0.033 (3)	0.013 (2)	0.024 (3)	−0.0005 (17)	0.005 (2)	0.0048 (17)
C13	0.019 (2)	0.026 (2)	0.022 (2)	−0.0073 (18)	0.0077 (17)	−0.0017 (19)
C14	0.014 (2)	0.022 (2)	0.028 (3)	−0.0019 (18)	−0.0042 (17)	0.000 (2)
C15	0.017 (2)	0.0137 (19)	0.021 (2)	−0.0018 (17)	−0.0030 (16)	0.0028 (17)
C16	0.013 (2)	0.025 (2)	0.026 (3)	−0.0034 (18)	0.0031 (17)	−0.0086 (19)
C17	0.019 (2)	0.029 (2)	0.027 (3)	−0.0072 (19)	0.0046 (18)	−0.011 (2)
C18	0.028 (3)	0.034 (2)	0.020 (3)	−0.018 (2)	0.0068 (18)	−0.005 (2)
C19	0.032 (3)	0.020 (2)	0.023 (3)	−0.011 (2)	0.0017 (19)	0.0021 (19)
C20	0.024 (2)	0.018 (2)	0.020 (2)	−0.0042 (18)	−0.0023 (17)	−0.0009 (19)
C21	0.018 (2)	0.015 (2)	0.017 (2)	−0.0063 (17)	0.0012 (16)	−0.0051 (18)
C22	0.015 (2)	0.013 (2)	0.030 (3)	−0.0002 (17)	−0.0009 (17)	−0.0014 (18)
C23	0.019 (2)	0.017 (2)	0.016 (2)	0.0020 (17)	−0.0036 (16)	−0.0025 (17)
C24	0.015 (2)	0.010 (2)	0.027 (3)	−0.0047 (16)	−0.0021 (16)	0.0000 (17)
C25	0.0101 (19)	0.017 (2)	0.018 (2)	0.0042 (16)	−0.0041 (15)	−0.0021 (17)
C26	0.029 (3)	0.019 (2)	0.024 (3)	0.0003 (18)	−0.0016 (19)	0.0010 (19)
C27	0.037 (3)	0.024 (2)	0.020 (3)	0.007 (2)	−0.0065 (19)	−0.010 (2)
C28	0.030 (3)	0.033 (3)	0.013 (2)	0.011 (2)	−0.0030 (17)	0.002 (2)
C29	0.028 (3)	0.022 (2)	0.025 (3)	0.0038 (19)	0.0007 (18)	0.0072 (19)
C30	0.022 (2)	0.019 (2)	0.020 (2)	0.0023 (17)	−0.0032 (17)	0.0005 (18)
C31	0.025 (2)	0.020 (2)	0.033 (3)	−0.0095 (18)	−0.0069 (18)	0.001 (2)
C32	0.013 (2)	0.017 (2)	0.024 (3)	−0.0030 (17)	0.0025 (16)	−0.0024 (18)
C33	0.021 (2)	0.021 (2)	0.018 (2)	−0.0076 (17)	−0.0081 (16)	−0.0038 (18)
C34	0.017 (2)	0.021 (2)	0.017 (2)	0.0016 (16)	−0.0033 (17)	0.0028 (17)
C35	0.027 (2)	0.022 (2)	0.027 (3)	−0.0014 (18)	−0.0109 (18)	0.0031 (19)

### Geometric parameters (Å, °)

**Table:**

Ir1—C9	2.000 (4)	C14—C15	1.387 (5)
Ir1—C24	1.988 (4)	C14—H14	0.9300
Ir1—N1	2.045 (3)	C15—H15	0.9300
Ir1—N2	2.049 (3)	C16—C17	1.380 (5)
Ir1—O1	2.137 (2)	C16—C21	1.411 (5)
Ir1—O2	2.137 (3)	C17—C18	1.385 (6)
S1—C7	1.739 (4)	C17—H17	0.9300
S1—C1	1.743 (4)	C18—C19	1.407 (6)
S2—C22	1.734 (4)	C18—H18	0.9300
S2—C16	1.746 (4)	C19—C20	1.373 (5)
F1—C13	1.376 (4)	C19—H19	0.9300
F2—C28	1.368 (5)	C20—C21	1.393 (6)
O1—C32	1.273 (5)	C20—H20	0.9300
O2—C34	1.280 (5)	C22—C23	1.416 (5)
N1—C7	1.348 (4)	C23—C24	1.372 (5)
N1—C6	1.386 (5)	C23—H23	0.9300
N2—C22	1.336 (5)	C24—C25	1.477 (5)
N2—C21	1.382 (5)	C25—C30	1.384 (5)
C1—C2	1.399 (5)	C25—C26	1.394 (5)
C1—C6	1.399 (5)	C26—C27	1.384 (6)
C2—C3	1.389 (6)	C26—H26	0.9300
C2—H2	0.9300	C27—C28	1.375 (6)
C3—C4	1.391 (6)	C27—H27	0.9300
C3—H3	0.9300	C28—C29	1.369 (6)
C4—C5	1.385 (5)	C29—C30	1.388 (5)
C4—H4	0.9300	C29—H29	0.9300
C5—C6	1.391 (5)	C30—H30	0.9300
C5—H5	0.9300	C31—C32	1.518 (5)
C7—C8	1.404 (5)	C31—H31A	0.9600
C8—C9	1.371 (5)	C31—H31B	0.9600
C8—H8	0.9300	C31—H31C	0.9600
C9—C10	1.486 (5)	C32—C33	1.397 (5)
C10—C15	1.396 (5)	C33—C34	1.399 (5)
C10—C11	1.399 (5)	C33—H33	0.9300
C11—C12	1.380 (5)	C34—C35	1.505 (5)
C11—H11	0.9300	C35—H35A	0.9600
C12—C13	1.369 (6)	C35—H35B	0.9600
C12—H12	0.9300	C35—H35C	0.9600
C13—C14	1.378 (6)		
			
C24—Ir1—C9	98.48 (15)	C14—C15—H15	119.3
C24—Ir1—N1	96.20 (14)	C10—C15—H15	119.3
C9—Ir1—N1	80.09 (13)	C17—C16—C21	121.4 (4)
C24—Ir1—N2	80.01 (14)	C17—C16—S2	128.9 (3)
C9—Ir1—N2	97.92 (13)	C21—C16—S2	109.7 (3)
N1—Ir1—N2	175.46 (12)	C16—C17—C18	118.7 (4)
C24—Ir1—O2	173.43 (12)	C16—C17—H17	120.6
C9—Ir1—O2	87.76 (12)	C18—C17—H17	120.6
N1—Ir1—O2	86.88 (11)	C17—C18—C19	120.2 (4)
N2—Ir1—O2	97.15 (11)	C17—C18—H18	119.9
C24—Ir1—O1	85.77 (12)	C19—C18—H18	119.9
C9—Ir1—O1	175.04 (12)	C20—C19—C18	121.1 (4)
N1—Ir1—O1	96.96 (10)	C20—C19—H19	119.4
N2—Ir1—O1	85.30 (10)	C18—C19—H19	119.4
O2—Ir1—O1	88.10 (10)	C19—C20—C21	119.3 (4)
C7—S1—C1	90.17 (18)	C19—C20—H20	120.4
C22—S2—C16	90.42 (18)	C21—C20—H20	120.4
C32—O1—Ir1	125.0 (2)	N2—C21—C20	127.4 (4)
C34—O2—Ir1	124.9 (2)	N2—C21—C16	113.3 (4)
C7—N1—C6	113.1 (3)	C20—C21—C16	119.3 (4)
C7—N1—Ir1	112.0 (2)	N2—C22—C23	118.1 (3)
C6—N1—Ir1	134.8 (2)	N2—C22—S2	113.3 (3)
C22—N2—C21	113.3 (3)	C23—C22—S2	128.1 (3)
C22—N2—Ir1	111.7 (3)	C24—C23—C22	113.6 (4)
C21—N2—Ir1	135.0 (3)	C24—C23—H23	123.2
C2—C1—C6	121.4 (4)	C22—C23—H23	123.2
C2—C1—S1	128.0 (3)	C23—C24—C25	120.1 (4)
C6—C1—S1	110.6 (3)	C23—C24—Ir1	114.6 (3)
C3—C2—C1	117.8 (4)	C25—C24—Ir1	124.9 (3)
C3—C2—H2	121.1	C30—C25—C26	118.8 (4)
C1—C2—H2	121.1	C30—C25—C24	120.3 (3)
C2—C3—C4	120.4 (4)	C26—C25—C24	120.9 (3)
C2—C3—H3	119.8	C27—C26—C25	120.4 (4)
C4—C3—H3	119.8	C27—C26—H26	119.8
C5—C4—C3	122.2 (4)	C25—C26—H26	119.8
C5—C4—H4	118.9	C28—C27—C26	118.7 (4)
C3—C4—H4	118.9	C28—C27—H27	120.7
C4—C5—C6	117.9 (4)	C26—C27—H27	120.7
C4—C5—H5	121.1	F2—C28—C29	118.9 (4)
C6—C5—H5	121.1	F2—C28—C27	118.3 (4)
N1—C6—C5	126.5 (3)	C29—C28—C27	122.8 (4)
N1—C6—C1	113.1 (3)	C28—C29—C30	117.7 (4)
C5—C6—C1	120.3 (3)	C28—C29—H29	121.1
N1—C7—C8	117.8 (3)	C30—C29—H29	121.1
N1—C7—S1	112.9 (3)	C25—C30—C29	121.5 (4)
C8—C7—S1	129.0 (3)	C25—C30—H30	119.2
C9—C8—C7	114.6 (3)	C29—C30—H30	119.2
C9—C8—H8	122.7	C32—C31—H31A	109.5
C7—C8—H8	122.7	C32—C31—H31B	109.5
C8—C9—C10	119.9 (3)	H31A—C31—H31B	109.5
C8—C9—Ir1	114.2 (3)	C32—C31—H31C	109.5
C10—C9—Ir1	125.7 (3)	H31A—C31—H31C	109.5
C15—C10—C11	118.2 (3)	H31B—C31—H31C	109.5
C15—C10—C9	121.4 (3)	O1—C32—C33	126.7 (3)
C11—C10—C9	120.4 (3)	O1—C32—C31	114.5 (3)
C12—C11—C10	121.2 (4)	C33—C32—C31	118.7 (4)
C12—C11—H11	119.4	C32—C33—C34	127.5 (4)
C10—C11—H11	119.4	C32—C33—H33	116.2
C13—C12—C11	118.3 (4)	C34—C33—H33	116.2
C13—C12—H12	120.8	O2—C34—C33	126.5 (4)
C11—C12—H12	120.8	O2—C34—C35	114.6 (3)
C12—C13—F1	118.4 (4)	C33—C34—C35	118.8 (3)
C12—C13—C14	123.3 (3)	C34—C35—H35A	109.5
F1—C13—C14	118.3 (4)	C34—C35—H35B	109.5
C13—C14—C15	117.5 (4)	H35A—C35—H35B	109.5
C13—C14—H14	121.2	C34—C35—H35C	109.5
C15—C14—H14	121.2	H35A—C35—H35C	109.5
C14—C15—C10	121.5 (4)	H35B—C35—H35C	109.5
			
C24—Ir1—O1—C32	−167.6 (3)	Ir1—C9—C10—C11	130.0 (3)
C9—Ir1—O1—C32	43.4 (15)	C15—C10—C11—C12	0.9 (6)
N1—Ir1—O1—C32	96.7 (3)	C9—C10—C11—C12	179.9 (4)
N2—Ir1—O1—C32	−87.3 (3)	C10—C11—C12—C13	−2.3 (6)
O2—Ir1—O1—C32	10.0 (3)	C11—C12—C13—F1	−178.4 (4)
C24—Ir1—O2—C34	11.2 (12)	C11—C12—C13—C14	2.7 (6)
C9—Ir1—O2—C34	172.9 (3)	C12—C13—C14—C15	−1.6 (6)
N1—Ir1—O2—C34	−106.9 (3)	F1—C13—C14—C15	179.5 (3)
N2—Ir1—O2—C34	75.2 (3)	C13—C14—C15—C10	0.1 (6)
O1—Ir1—O2—C34	−9.9 (3)	C11—C10—C15—C14	0.3 (6)
C24—Ir1—N1—C7	106.5 (3)	C9—C10—C15—C14	−178.7 (4)
C9—Ir1—N1—C7	9.0 (3)	C22—S2—C16—C17	179.6 (4)
N2—Ir1—N1—C7	73.3 (14)	C22—S2—C16—C21	0.4 (3)
O2—Ir1—N1—C7	−79.3 (3)	C21—C16—C17—C18	0.7 (6)
O1—Ir1—N1—C7	−167.0 (2)	S2—C16—C17—C18	−178.5 (3)
C24—Ir1—N1—C6	−72.7 (4)	C16—C17—C18—C19	0.3 (6)
C9—Ir1—N1—C6	−170.3 (4)	C17—C18—C19—C20	−0.7 (6)
N2—Ir1—N1—C6	−106.0 (13)	C18—C19—C20—C21	0.1 (6)
O2—Ir1—N1—C6	101.4 (4)	C22—N2—C21—C20	−177.6 (4)
O1—Ir1—N1—C6	13.7 (4)	Ir1—N2—C21—C20	2.7 (6)
C24—Ir1—N2—C22	11.8 (3)	C22—N2—C21—C16	1.5 (5)
C9—Ir1—N2—C22	109.1 (3)	Ir1—N2—C21—C16	−178.2 (3)
N1—Ir1—N2—C22	45.4 (15)	C19—C20—C21—N2	179.9 (4)
O2—Ir1—N2—C22	−162.2 (2)	C19—C20—C21—C16	0.8 (6)
O1—Ir1—N2—C22	−74.7 (2)	C17—C16—C21—N2	179.6 (3)
C24—Ir1—N2—C21	−168.5 (4)	S2—C16—C21—N2	−1.1 (4)
C9—Ir1—N2—C21	−71.2 (4)	C17—C16—C21—C20	−1.2 (6)
N1—Ir1—N2—C21	−134.9 (13)	S2—C16—C21—C20	178.1 (3)
O2—Ir1—N2—C21	17.5 (3)	C21—N2—C22—C23	171.1 (3)
O1—Ir1—N2—C21	105.0 (3)	Ir1—N2—C22—C23	−9.1 (4)
C7—S1—C1—C2	179.4 (4)	C21—N2—C22—S2	−1.2 (4)
C7—S1—C1—C6	−0.3 (3)	Ir1—N2—C22—S2	178.58 (16)
C6—C1—C2—C3	1.2 (6)	C16—S2—C22—N2	0.5 (3)
S1—C1—C2—C3	−178.4 (3)	C16—S2—C22—C23	−170.9 (4)
C1—C2—C3—C4	−0.9 (6)	N2—C22—C23—C24	−1.5 (5)
C2—C3—C4—C5	0.1 (7)	S2—C22—C23—C24	169.5 (3)
C3—C4—C5—C6	0.4 (6)	C22—C23—C24—C25	−174.8 (3)
C7—N1—C6—C5	−178.1 (4)	C22—C23—C24—Ir1	11.9 (4)
Ir1—N1—C6—C5	1.2 (6)	C9—Ir1—C24—C23	−109.6 (3)
C7—N1—C6—C1	1.4 (5)	N1—Ir1—C24—C23	169.6 (3)
Ir1—N1—C6—C1	−179.3 (3)	N2—Ir1—C24—C23	−13.0 (3)
C4—C5—C6—N1	179.3 (4)	O2—Ir1—C24—C23	51.9 (12)
C4—C5—C6—C1	−0.1 (6)	O1—Ir1—C24—C23	73.0 (3)
C2—C1—C6—N1	179.7 (4)	C9—Ir1—C24—C25	77.5 (3)
S1—C1—C6—N1	−0.5 (5)	N1—Ir1—C24—C25	−3.4 (3)
C2—C1—C6—C5	−0.7 (6)	N2—Ir1—C24—C25	174.1 (3)
S1—C1—C6—C5	179.0 (3)	O2—Ir1—C24—C25	−121.1 (10)
C6—N1—C7—C8	172.9 (3)	O1—Ir1—C24—C25	−99.9 (3)
Ir1—N1—C7—C8	−6.6 (4)	C23—C24—C25—C30	−62.4 (5)
C6—N1—C7—S1	−1.7 (4)	Ir1—C24—C25—C30	110.2 (3)
Ir1—N1—C7—S1	178.90 (17)	C23—C24—C25—C26	115.4 (4)
C1—S1—C7—N1	1.1 (3)	Ir1—C24—C25—C26	−72.1 (4)
C1—S1—C7—C8	−172.6 (4)	C30—C25—C26—C27	1.9 (6)
N1—C7—C8—C9	−1.9 (5)	C24—C25—C26—C27	−175.9 (4)
S1—C7—C8—C9	171.6 (3)	C25—C26—C27—C28	−1.8 (6)
C7—C8—C9—C10	−175.3 (3)	C26—C27—C28—F2	178.8 (4)
C7—C8—C9—Ir1	9.7 (5)	C26—C27—C28—C29	−0.7 (6)
C24—Ir1—C9—C8	−105.1 (3)	F2—C28—C29—C30	−176.5 (3)
N1—Ir1—C9—C8	−10.2 (3)	C27—C28—C29—C30	2.9 (6)
N2—Ir1—C9—C8	173.9 (3)	C26—C25—C30—C29	0.4 (6)
O2—Ir1—C9—C8	77.0 (3)	C24—C25—C30—C29	178.2 (3)
O1—Ir1—C9—C8	43.7 (16)	C28—C29—C30—C25	−2.8 (6)
C24—Ir1—C9—C10	80.3 (3)	Ir1—O1—C32—C33	−5.2 (6)
N1—Ir1—C9—C10	175.1 (3)	Ir1—O1—C32—C31	176.5 (2)
N2—Ir1—C9—C10	−0.8 (3)	O1—C32—C33—C34	−4.9 (7)
O2—Ir1—C9—C10	−97.7 (3)	C31—C32—C33—C34	173.4 (4)
O1—Ir1—C9—C10	−131.0 (13)	Ir1—O2—C34—C33	4.8 (6)
C8—C9—C10—C15	134.5 (4)	Ir1—O2—C34—C35	−175.1 (2)
Ir1—C9—C10—C15	−51.1 (5)	C32—C33—C34—O2	5.1 (7)
C8—C9—C10—C11	−44.4 (5)	C32—C33—C34—C35	−175.0 (4)
